# Supplementary material for: IFN-γ stimulated murine and human neurons mount anti-parasitic defenses against the intracellular parasite Toxoplasma gondii
Source: Nat Commun. 2022 Aug 8;13:4605. doi: 10.1038/s41467-022-32225-z (PMC9360015; doi:10.1038/s41467-022-32225-z)
Supplement: Supplementary file 1 — Supplementary information [file 41467_2022_32225_MOESM1_ESM.pdf]

Supplementary Information for IFN-γ stimulated murine and human neurons mount anti-parasitic defenses against the intracellular parasite *Toxoplasma gondii*

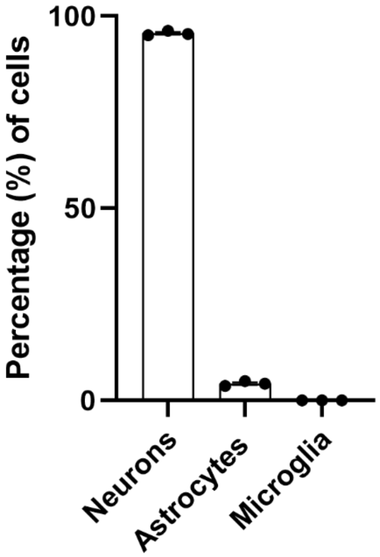

Supplementary Figure 1. *In vitro* neuronal cultures show little glial contamination Quantification of different CNS cell types in primary murine neuron cultures. At 12 DIV, cultures were fixed and stained with anti-Tuj1 antibodies (neurons), an anti-astrocyte cocktail (anti-GFAP, anti-S100B, anti-ALDH1L1 antibodies), and anti-Iba1 antibodies (microglia). The fixed and stained cultures were then analyzed by epifluorescent microscopy. Bars, mean ± SEM. N = 100 cells counted/experiment, 3 independent experiments. Source data are provided as a Source Data file.

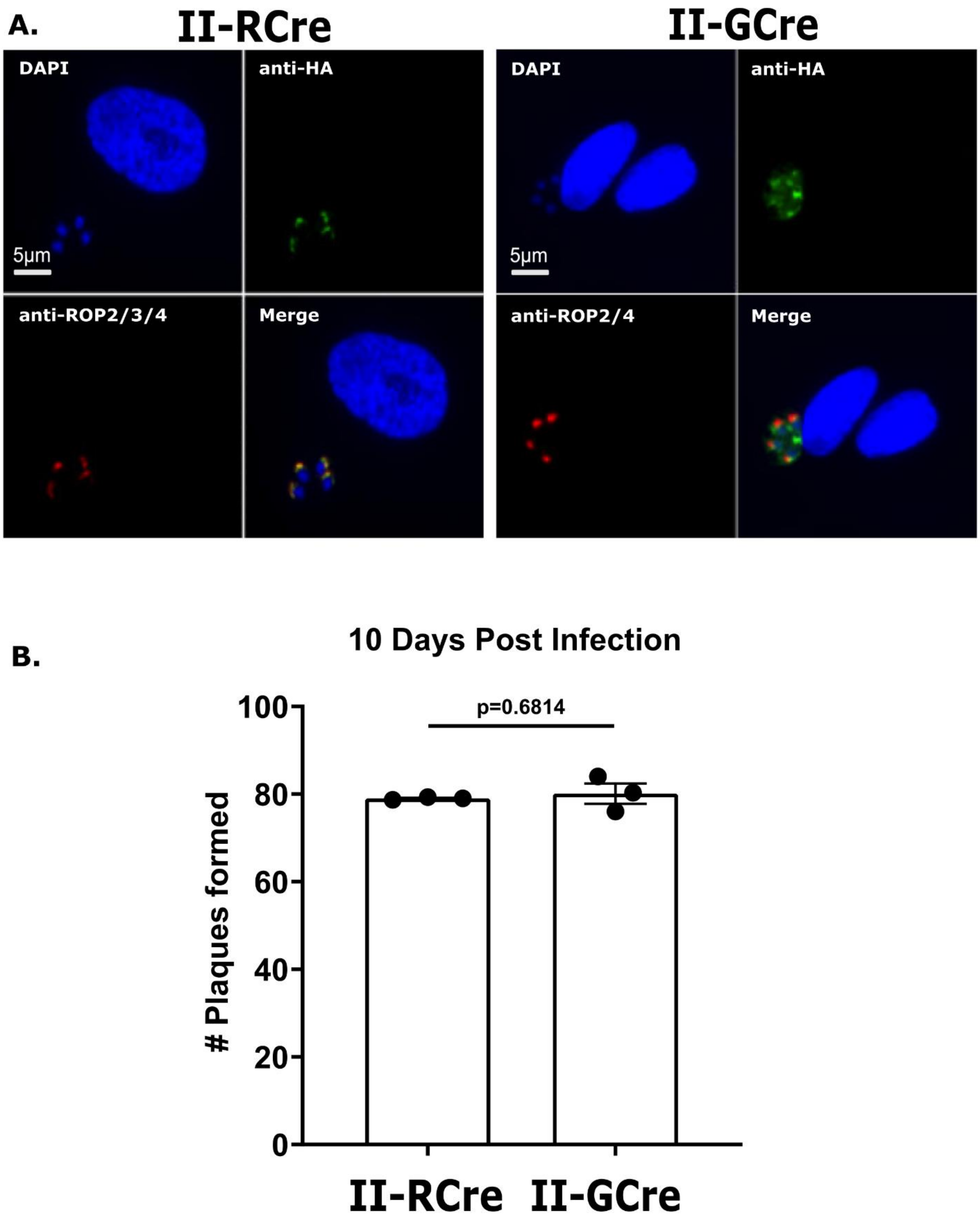
A.
II-RCre
II-GCre
DAPI
anti-HA
5µm
anti-ROP2/3/4
Merge
DAPI
anti-HA
5µm
anti-ROP2/4
Merge
B.
10 Days Post Infection
p=0.6814
# Plaques formed
100
80
60
40
20
0
II-RCre
II-GCre

Supplementary Figure 2. GCre expression does not affect parasite viability.

A. Representative images of immunofluorescent assays with parasites that express HA-tagged RCre or GCre fusion proteins. HFFs were infected with identified strains (MOI=1) for 24 hours, after which the cultures were fixed and stained with anti-HA antibodies, anti-ROP2/4 antibodies, and DAPI. Green = anti-HA antibodies, red = anti-ROP2/4 antibodies, and blue = DAPI. **B.** Quantification of plaque assays. HFFs were infected with 200 parasites of the indicated strains. At 10 dpi, the cultures were fixed, stained with Cresyl violet, and plaques counted. Bars, mean ± SEM. Each dot = 1 experiment, N = 3 independent experiments. Unpaired t-test with Welch's t correction. Source data are provided as a Source Data file.

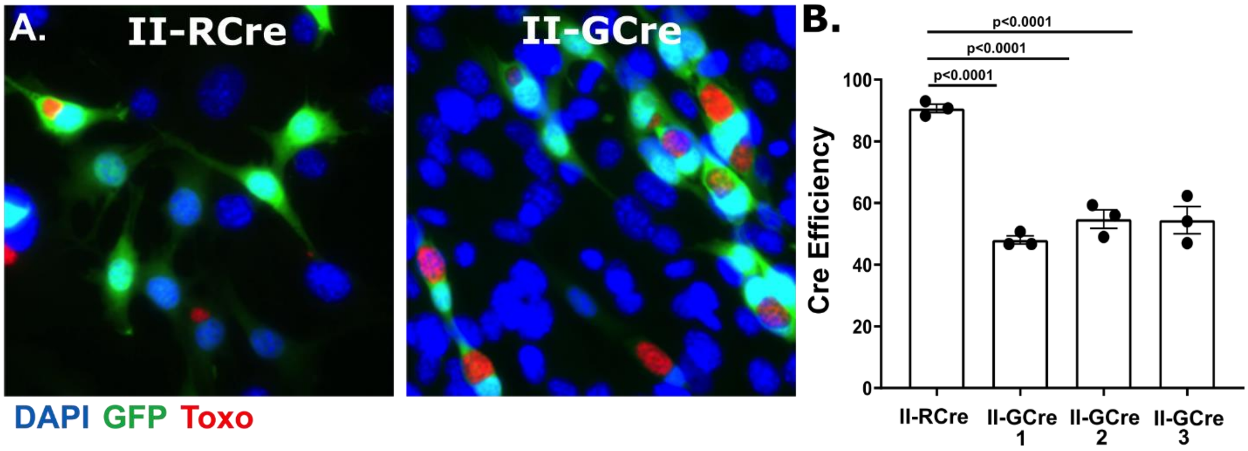

Supplementary Figure 3. II-GCre is capable of Cre-mediated recombination

Cre reporter fibroblast that express GFP only after Cre-mediated recombination were infected with identified strains (MOI 1) for 24 hours and then fixed and stained with anti-SAG1 antibodies and DAPI. Green = GFP; red = anti-SAG1 antibodies; and blue = DAPI. **A.** Representative merged images of infected Cre reporter fibroblasts cultures wit listed strains. **B.** Quantification of Cre-mediated recombination efficiency. Cre reporter fibroblasts were infected with II-RCre parasites or 3 different II-GCre clones. Efficiency at triggering Cre-mediated recombination was quantified by dividing the number of GFP^{+} $parasite^{+}$ cells by the total number of $parasite^{+}$ cells multiplied by 100. Bars, mean ± SEM. N = 100 infected cells/experiment, 3 experiments/strain or clone. One-way ANOVA with Dunnett's multiple comparison test. Source data are provided as a Source Data file.

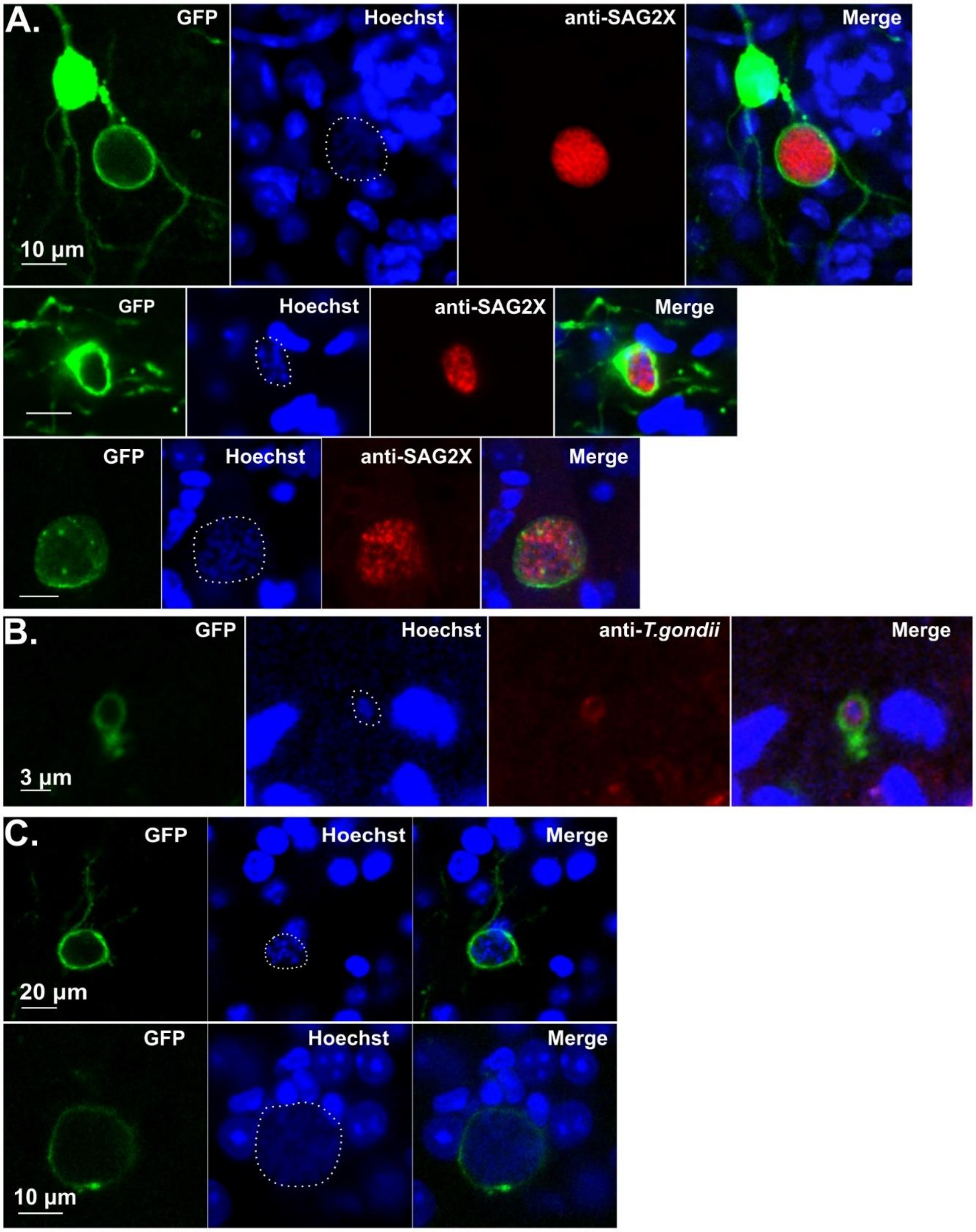

Supplementary Figure 4. Hoechst staining of *T. gondii* nuclei coincides with anti-*T. gondii* antibody staining. To confirm that Hoechst$^+$ DNA found within GFP$^+$

neurons identified *T. gondii* nuclei, PACT cleared 200 μm thick brain sections were stained with either of two anti-*T. gondii* antibodies (mouse anti-SAG2X or rabbit polyclonal anti-*T. gondii*), followed by imaging on a confocal microscope and rendering using Imaris software. Panels **A-C** show individual slices of the z-stacks. Green = GFP, blue = Hoechst, and red = anti-*T. gondii* antibodies. White dotted circles in Hoechst panel identifies stained parasite DNA. **A.** Representative images of 3 different infected GFP^+ neurons from sections stained with anti-SAG2X antibodies. Note how GFP is excluded from the parasitophorous vacuole (PV). Scale bar 10 μm. Six of 18 *T. gondii*$^+$$GFP^+$ neurons were confirmed with anti-SAG2X antibody staining. **B.** Representative images of a GFP^+ neuron from a section stained with anti-*T. gondii* antibodies. Note that this PV contains only one parasite. Eight of 18 *T. gondii*$^+$$GFP^+$ neurons were confirmed with anti-*T. gondii* antibody staining **C.** Representative images of two more infected GFP^+ neurons, where GFP exclusion and Hoechst are used to identify parasite infected neurons. Four of 18 *T. gondii*$^+$$GFP^+$ neurons were confirmed by Hoechst staining only.

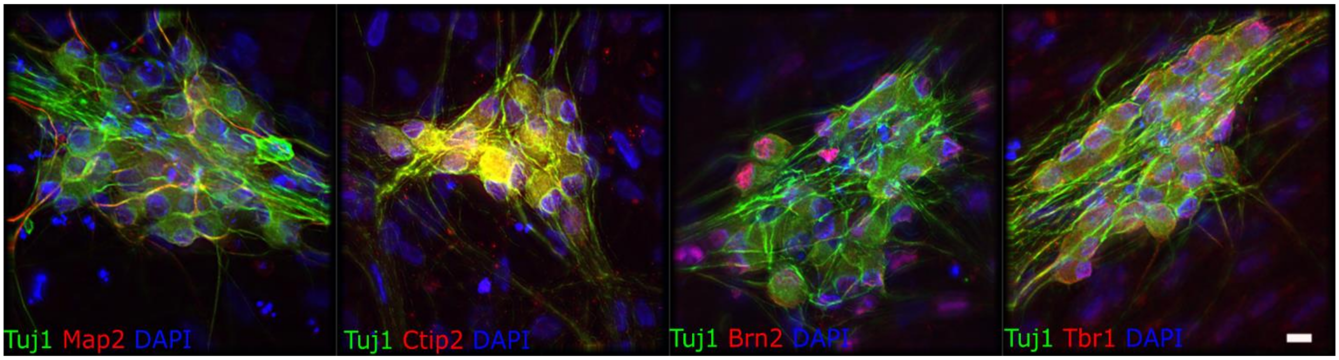

Supplementary Figure 5. Characterization of human neurons for cortical layer markers

Representative images of hPSC neurons stained with different markers for mature cortical neurons. HPSC neurons were grown and differentiated for 14 days, after which cultures were fixed and stained with DAPI (blue), anti-Tuj1 antibodies (pan neuronal marker, green) and one of the following (red): anti-Map2 antibodies (global mature neuronal marker), anti-Ctip2 antibodies (cortical layer V, VI), anti-Brn2 antibodies (cortical layer II-V), or anti-Tbr1 antibodies (cortical layer I, V, VI). Scale bar = 25µm. The staining for the markers were performed for neurons from two independent differentiations.

Supplementary Table 1. Primers used in this paper

Gene name	Primer sequence
**STAT1**	FP: AAAGTCATGGCTGCCGAGAA<br>RP: CATCGGTTCTGGTGCTTCCT
**IRF1**	FP: ATCTCGGGCATCTTTCGCTT<br>RP: TGATTGGCATGGTGGCTTTG
**Irgm1**	FP: CATAGGGAACTTCTGCCGGA<br>RP: ATACTCCTCAAACCCTGATCCA
**Gbp2**	FP: AGCTGCTAAACTTCGGGAACA<br>RP: GCCTTGGGCCTTCAGAGTAT
**Irgb6**	FP: GAGCTTCTACCATACAGAGCCATG<br>RP: CACTCTCGATGTCTCTCAGTACC
**Irga6**	FP: GTGCTCAATGTTGCTGTCACC<br>RP: CACCCCAGTTTTAGCTGCAC
**MHCI**	FP: CAAGAGCAGTGGTTCCGAGTGAG<br>RP: TTTTCAGGTCTTCGTTCAGGGCG
**Cre CDS**	FP: GGCGCCCCCAAGAAGAAG<br>RP: GATCAGCACGAAACCTTGC
**Gra16 CDS**	FP: GGTTTCGTGCTGATCGCCGTTCATTGCATTCAGAATACG<br>RP: CTTCTTGGGGGCGCCCATCTGATCATTTTTCCGCTTCGC
