# Supplementary material for: IFN-γ stimulated murine and human neurons mount anti-parasitic defenses against the intracellular parasite Toxoplasma gondii
Source: Nat Commun. 2022 Aug 8;13:4605. doi: 10.1038/s41467-022-32225-z (PMC9360015; doi:10.1038/s41467-022-32225-z)
Supplement: Supplementary file 3 — Reporting Summary [file 41467_2022_32225_MOESM3_ESM.pdf]

# nature portfolio

Corresponding author(s): Anita A Koshy

Last updated by author(s): Jul 13, 2022



# Reporting Summary

Nature Portfolio wishes to improve the reproducibility of the work that we publish. This form provides structure for consistency and transparency in reporting. For further information on Nature Portfolio policies, see our Editorial Policies and the Editorial Policy Checklist.

## Statistics

For all statistical analyses, confirm that the following items are present in the figure legend, table legend, main text, or Methods section.

| n/a | Confirmed | |
|---|---|---|
| ☐ | ☒ | The exact sample size (*n*) for each experimental group/condition, given as a discrete number and unit of measurement |
| ☐ | ☒ | A statement on whether measurements were taken from distinct samples or whether the same sample was measured repeatedly |
| ☐ | ☒ | The statistical test(s) used AND whether they are one- or two-sided<br>*Only common tests should be described solely by name; describe more complex techniques in the Methods section.* |
| ☒ | ☐ | A description of all covariates tested |
| ☐ | ☒ | A description of any assumptions or corrections, such as tests of normality and adjustment for multiple comparisons |
| ☐ | ☒ | A full description of the statistical parameters including central tendency (e.g. means) or other basic estimates (e.g. regression coefficient) AND variation (e.g. standard deviation) or associated estimates of uncertainty (e.g. confidence intervals) |
| ☐ | ☒ | For null hypothesis testing, the test statistic (e.g. *F*, *t*, *r*) with confidence intervals, effect sizes, degrees of freedom and *P* value noted<br>*Give P values as exact values whenever suitable.* |
| ☒ | ☐ | For Bayesian analysis, information on the choice of priors and Markov chain Monte Carlo settings |
| ☒ | ☐ | For hierarchical and complex designs, identification of the appropriate level for tests and full reporting of outcomes |
| ☒ | ☐ | Estimates of effect sizes (e.g. Cohen's *d*, Pearson's *r*), indicating how they were calculated |

*Our web collection on statistics for biologists contains articles on many of the points above.*

## Software and code

Policy information about availability of computer code

| | |
|---|---|
| Data collection | No code was used in data collection. 3D rendering (IMARIS Software X64; version 9.7.2); Confocal imaging (Zen blue edition, version 2.6) |
| Data analysis | GraphPad Prism (GraphPad software; version 9.1.2); Image J (NIH, version 1.52K); Western blot software (Image Studio, version 5.2) |

For manuscripts utilizing custom algorithms or software that are central to the research but not yet described in published literature, software must be made available to editors and reviewers. We strongly encourage code deposition in a community repository (e.g. GitHub). See the Nature Portfolio guidelines for submitting code & software for further information.

## Data

Policy information about availability of data

All manuscripts must include a data availability statement. This statement should provide the following information, where applicable:

- Accession codes, unique identifiers, or web links for publicly available datasets
- A description of any restrictions on data availability
- For clinical datasets or third party data, please ensure that the statement adheres to our policy

No large datasets that require deposition in public databases were generated in this manuscript. We have provided source data for Figures 1-6 and supplemental figures 1-3.

## Human research participants

Policy information about studies involving human research participants and Sex and Gender in Research.

| | |
|---|---|
| Reporting on sex and gender | n/a |
| Population characteristics | n/a |
| Recruitment | n/a |
| Ethics oversight | n/a |

Note that full information on the approval of the study protocol must also be provided in the manuscript.

# Field-specific reporting

Please select the one below that is the best fit for your research. If you are not sure, read the appropriate sections before making your selection.

- [x] Life sciences
- [ ] Behavioural & social sciences
- [ ] Ecological, evolutionary & environmental sciences

For a reference copy of the document with all sections, see nature.com/documents/nr-reporting-summary-flat.pdf

# Life sciences study design

All studies must disclose on these points even when the disclosure is negative.

| | |
|---|---|
| Sample size | Pilot studied determined that the 24+ hour time point for neuronal infection required low MOIs (and infection rates). Low infection rates leads to high variability, which was addressed by analyzing high numbers of neurons (750-1000). For other experiments (Irga6+ or GFP+ neuron rates of infection), we chose the sample size using what was feasible while still ensuring we were sampling relatively large number (100-200 events). |
| Data exclusions | No data were excluded from the analysis presented in the study |
| Replication | At least three independent biological replicates were performed for every experiment except for experiments in human neurons. In these experiments, consistent with what is standard in this field, we used two-independent differentiations with 2 technical replicates for each differentiation. For the whole neuron reconstructions, we consider each mouse a "replicate". No replicates failed. |
| Randomization | Randomization is incredibly important for clinical trials where each human is unique. Given that we were primarily working in cell culture, it's difficult to imagine how one truly "randomizes" the wells while still maintaining good data integrity. |
| Blinding | For all imaging analysis (relevant to Fig 1D; 2A-C, F, H; 3B, D, F, H; 4A, B, D; 6B, D; Supp Figs 2B, 3B) the investigator was blinded to group allocation during data collection. Once all data were collected, the investigator was unblinded to run statistical tests. Blinding was not done for Q-PCR or western blots- I will have to think about how we would do that. For neuron reconstructions, there is no way to blind the investigator since she is constructing a neuron and then determining whether or not she sees parasites within that neuron. |

# Reporting for specific materials, systems and methods

We require information from authors about some types of materials, experimental systems and methods used in many studies. Here, indicate whether each material, system or method listed is relevant to your study. If you are not sure if a list item applies to your research, read the appropriate section before selecting a response.

## Materials & experimental systems

| n/a | Involved in the study | |
|---|---|---|
| [ ] | [x] | Antibodies |
| [ ] | [x] | Eukaryotic cell lines |
| [x] | [ ] | Palaeontology and archaeology |
| [ ] | [x] | Animals and other organisms |
| [x] | [ ] | Clinical data |
| [x] | [ ] | Dual use research of concern |

## Methods

| n/a | Involved in the study | |
|---|---|---|
| [x] | [ ] | ChIP-seq |
| [x] | [ ] | Flow cytometry |
| [x] | [ ] | MRI-based neuroimaging |

## Antibodies

Antibodies used

The following primary antibodies were used in the study: mouse anti-tubulin beta III isoform (Tuj1),clone TU20 (MAB1637, Millipore, 1:1000); rabbit anti- β3-Tubulin, D71G9 (similar to Tuj1) (5568S, CST, 1:1000); mouse anti-NeuN clone A60 (MAB377, Millipore, 1:1000); rabbit anti-Glial Fibrillary Acidic protein (GFAP) (Z0334, DAKO, 1:500); rabbit anti S100 (Z0311, DAKO, 1:500); rabbit anti-ALDH1L1 (Ab87117, Abcam, 1:500); chicken anti-Iba1 (Ab 139590, Abcam, 1:500), rabbit anti-STAT1 (Ab47425, Abcam; 1:500); mouse anti-pSTAT1 pY701 clone14/p-STAT1 (612132, BD Biosciences, 1:250); rabbit anti-pSTAT1 Tyr701, Clone 58D6 (9167, CST, 1:200); mouse anti-SAG1 DG52 (gift John Boothroyd, 1:10,000); mouse anti-SRS-9 (gift John Boothroyd, 1:10,000); mouse anti-Irga6 (1:1500), mouse anti-Irgb6 (1:250) (gift Jonathan Howard); rabbit ant-HA C29F4 (3724S, CST, 1:500); mouse anti-ROP2/4 (1:1000, gift John Boothroyd); DAPI (D3571, Thermo Fisher, 1:1000); Hoechst 33342 Trihydrochloride, Trihydrate (H3570, Thermo Fisher, 1:1000).

Validation

anti-SAG1 (ms) : Burg et al J Immunology 1988
anti-SRS-9 (ms): Kim, S. K., and J. C. Boothroyd.2005. Stage-specific expression of surface antigens by Toxoplasma gondii as a mechanism to facilitate parasite persistence. J. Immunol.174:8038-8048.
anti-Irga6 (ms): Khaminets, A. et al. Coordinated loading of IRG resistance GTPases on to the Toxoplasma gondii parasitophorous vacuole. Cell. Microbiol. 12, 939–961 (2010).
anti-Irgb6 (ms): Khaminets, A. et al. Coordinated loading of IRG resistance GTPases on to the Toxoplasma gondii parasitophorous vacuole. Cell. Microbiol. 12, 939–961 (2010).
anti-ROP2/4 (ms): Sadak A et al Molecular and Biochemical Parasitology 1988
anti-tubulin beta III isoform (ms): Choi et al. Validated for western blot and IFA: In vivo reprogrammed pluripotent stem cells from teratomas share analogous properties with their in vitro counterparts. Scientific reports 5 13559 2015.
rabbit anti- β3-Tubulin (rb): Validated for western blot and IFA: Yaoming Wang et al. 3K3A-activated protein C stimulates postischemic neuronal repair by human neural stem cells in mice. Nat Med 2016
anti-NeuN (ms): validated for IFA. Sharaf et al. Localization of reelin signaling pathway components in murine midbrain and striatum. Cell and Tissue reasearch 359 393-407 (2015).
anti-GFAP (rb): Validated for IFA. Griffin and Sheehan et al. REV-ERBα mediates complement expression and diurnal regulation of microglial synaptic phagocytosis. eLife 2020.
anti-S100 (rb): Validated for IFA. Murru et al. Astrocyte-specific deletion of the mitochondrial m-AAA protease reveals glial contribution to neurodegeneration. Glia 2019.
anti-ALDH1L1 (rb): Validated for IFA. Caramello et al. Dentate gyrus development requires a cortical hem-derived astrocytic scaffold. eLife 2021.
anti-Iba1 (chk): Validated for IFA. Hubbs et al. Accumulation of Ubiquitin and Sequestosome-1 Implicate Protein Damage in Diacetyl-Induced Cytotoxicity.The American Journal of Pathology 2016.
anti-STAT1 (rb): Validated for western and IFA. Farini et al. Defective dystrophic thymus determines degenerative changes in skeletal muscle. Nat Commun 2021.
anti-pSTAT1 (ms): Validated for western blot. Kirchmeyer et al. Systematic Transcriptional Profiling of Responses to STAT1- and STAT3-Activating Cytokines in Different Cancer Types. Journal of Molecular Biology 2020.
anti-pSTAT1 (rb): Validated for IFA. Pandey et al. CXCL10/CXCR3 signaling contributes to an inflammatory microenvironment and its blockade enhances progression of murine pancreatic precancerous lesions. eLife 2021.
anti-HA (rb): Validated for IFA. Bosso et al. Nuclear PYHIN proteins target the host transcription factor Sp1 thereby restricting HIV-1 in human macrophages and CD4+ T cells. PLoS Pathogens 2020.

## Eukaryotic cell lines

Policy information about cell lines and Sex and Gender in Research

Cell line source(s)

Human foreskin fibroblasts (HFFs, gift of John Boothroyd)
Neuroprogenitors stem cells generated from WiCell WA07 are purchased from the University of Arizona Induced Pluripotent Stem Cell Core. The UA iPSC core obtained the WiCell WA07 line from WiCell Research Institute.
T. gondii: All T. gondii strains are described in Koshy, A. A. et al. Toxoplasma co-opts host cells it does not invade. PLoS Pathog. 8, e1002825 (2012) or Cabral, C. M. et al. Neurons are the Primary Target Cell for the Brain-Tropic Intracellular Parasite Toxoplasma gondii. PLoS Pathog. 12, e1005447 (2016) except for the new Gra-Cre strain which is described in this paper.

Authentication

n/a

Mycoplasma contamination

All cell lines and parasites are tested every month for mycoplasma contamination

Commonly misidentified lines
(See ICLAC register)

n/a

## Animals and other research organisms

Policy information about studies involving animals; ARRIVE guidelines recommended for reporting animal research, and Sex and Gender in Research

Laboratory animals

Cre reporter mice (Madisen et al., 2010) (#007906) were originally purchased from Jackson Laboratories. Breeding pairs of Irgm1/m3-/- (Irgm1/3 KO) mice (Collazo et al., 2001) were generously provided by Greg Taylor (Duke University, Durham, NC).

Wild animals

no wild animals were used in this study

| | |
|---|---|
| Reporting on sex | Pregnant females were used to harvest primary neurons from embryos of both sexes. For neuron reconstructions, available mice were used resulting in 1 female mouse and 4 male mice. |
| Field-collected samples | no field collected samples were used in this study |
| Ethics oversight | All procedures and experiments were carried out in accordance with the Public Health Service Policy on Human Care and Use of Laboratory Animals and approved by the University of Arizona's Institutional Animal Care and Use Committee (#12-391). |

Note that full information on the approval of the study protocol must also be provided in the manuscript.
